# Patient-Reported Self-Care Behaviors, Self-Efficacy, and Their Associated Factors in Men and Women with Coronary Heart Disease

**DOI:** 10.3390/healthcare14050653

**Published:** 2026-03-04

**Authors:** Gideon Victor, Ercole Vellone, Erika Sivarajan Froelicher

**Affiliations:** 1 Department of Biomedicine and Prevention, Tor-Vergata University of Rome, 00133 Rome, Italy; ercole.vellone@uniroma2.it; 2 Shifa College of Nursing, Shifa Tameer-e-Millat University, Islamabad 44000, Pakistan; erika.froelicher@ucsf.edu; 3 Faculty of Nursing and Midwifery, Wroclaw Medical University, 50-345 Wroclaw, Poland; 4 Department of Physiological Nursing, School of Nursing, University of California, San Francisco, CA 94143, USA; 5 Department of Epidemiology & Biostatistics, School of Medicine, University of California, San Francisco, CA 94143, USA

**Keywords:** coronary heart disease, self-care, self-efficacy, sex, factors

## Abstract

**Highlights:**

**What are the main findings?**

- Self-care maintenance was adequate in men. Self-care monitoring and self-care management were inadequate for both sexes.
- Depression scores were higher in men, while anxiety scores were higher in women.

**What are the implications of the main findings?**

- Healthcare providers should enhance self-care education. Interventions must address sedentary lifestyles in women and cigarette smoking in men.
- Strategies for medication adherence and symptom recognition are essential. Mental health support should be integrated into care.

**Abstract:**

**Background/Objective**: Heart disease is the leading cause of death for men and women across most racial and ethnic groups. Effective self-care improves patient outcomes. This study aimed to examine self-care and its associated variables in men and women with coronary heart disease. **Methods**: This cross-sectional survey enrolled patients with coronary heart disease through convenience sampling. Data were collected via in-person interview, including sociodemographic variables (e.g., age and sex) and clinical variables (e.g., comorbidities). We also used the Charlson Comorbidity Index to measure comorbidity; the Patient Health Questionnaire-9 to measure depression; and Generalized Anxiety Disorder-7 to measure anxiety. Self-care was evaluated with the Self-care Coronary Heart Disease Inventory and Self-Care Self-Efficacy Scale. Descriptive and multivariate analyses were performed. This study adhered to the CROSS guidelines. **Results**: The sample comprised 354 patients (57.6% men and 42.4% women). Self-care monitoring and management scores were inadequate with women. Self-care self-efficacy scores were marginally adequate. Men had worse depression, comorbidities, and smoking, while women had higher anxiety and a sedentary lifestyle. Older age, low education, public transportation use, sedentary lifestyle, comorbidity, anxiety, and depression were associated with worse self-care, whereas being single and ambulance accessibility improved self-care. **Conclusions**: Self-care monitoring and self-care management scores were inadequate for both sexes. Depression and public transportation use were inversely associated with all self-care domains. Depression and anxiety screening should be included in routine practice. Healthcare providers should enhance self-care education for CHD patients. Interventions must address sedentary lifestyles in women and cigarette smoking in men.

## 1. Introduction

Heart disease is the leading cause of death for men and women across most racial and ethnic groups, with coronary heart disease (CHD) being the most common type of heart disease [1]. Approximately one in 20 adults aged 20 and older is affected by CHD [2]. CHD poses a substantial global burden, with low and middle-income countries facing significant challenges due to epidemiological transition in many regions. Low- and middle-income countries account for more than three-quarters of the worldwide CHD burden [3]. The pattern of CHD prevalence and its associated death rates differ markedly between countries [4]. The prevalence of CHD was the highest 22.3% among Tunisians [5] and the lowest 2.5% among Sudanese [6]. Although CHD prevalence is higher among men [7], women also bear a substantial CHD burden [8]. Men have a higher death rate than women globally [9]. Global estimates from the World Health Organization (WHO) report the highest number of deaths in China, India, Russia, Ukraine, the United States of America, Brazil, Pakistan, Japan, and Indonesia [10]. Heart diseases cause around 29% of all deaths in Pakistan [11].

Self-care has the potential to improve the quality of life, prognosis, and mortality rates [12]. Whereas lack of self-care results in recurrent myocardial infarction, increased burden, and deterioration of illness. WHO accentuates behavioral, environmental and lifestyle modification for cardiac diseases and risk prevention [3]. Recent studies in different countries have reported low self-care among patients with CHD [13,14]. A recent mapping review found cigarette smoking higher in men, while sedentary life, obesity and dyslipidemia in women [15]. Self-care is generically examined in previous research, considering only specific behaviors. In addition, there is a lack of studies focusing on sex-specific differences and preferences. Cultural norms may restrict women’s mobility and independence, limiting their ability to access healthcare services. The specific impact of sex on self-care in CHD remains uncertain [16], and this has not been routinely collected or translated into practice.

Despite significant advancements in the management of CHD, challenges persist in improving patient prognosis and enhancing quality of life. Recently, there has been a notable surge in the volume of literature delving into the impact of factors such as specific health issues, individual characteristics, and environment on self-care [17]. In this context, the Situation-Specific Theory of Heart Failure Self-care, even though focused on heart failure, identified person, problem, and environment-related variables influencing self-care [17], and self-care as a decision-making process applicable across cardiovascular conditions, including CHD. Indeed, in this condition, there could be person-related variables (e.g., sex, marital status, body mass index), problem-related variables (e.g., comorbidity, anxiety, and depression), and environment-related variables (e.g., residence, commuting mode) associated with self-care in CHD. Regional evidence on sex differences in self-care among patients with CHD remains limited. Furthermore, there is a paucity of studies that comprehensively examine the association between these multidimensional factors and self-care in CHD populations. This study aimed to examine self-care behaviors and their associated variables in men and women with CHD.

## 2. Materials and Methods

This study represents a cross-sectional analysis of baseline data derived from an ongoing longitudinal cohort of patients with CHD because the objective of this study was to examine the contemporaneous associations among variables. The longitudinal cohort study seeks to examine the impact of self-care behaviors, sociodemographic and clinical variables, on events within 90 days of hospital readmissions, emergency department visits, and unplanned clinic visits among patients with CHD. This study adhered to the CROSS guidelines [18].

### 2.1. Study Setting

This study was conducted in a private teaching hospital in Islamabad, Pakistan. The hospital is a renowned, internationally accredited tertiary care facility. The hospital has thirty-two dedicated beds for cardiac patients, a cardiac department, a cardiac unit, an intensive coronary care unit, and a cardiac catheterization laboratory.

### 2.2. Eligibility Criteria and Recruitment

The patients were ≥18 years old and had a documented diagnosis of CHD. Patients who met at least two of the three criteria for CHD (i.e., chest pain, enzyme changes, and electrocardiographic changes) [19], as determined through a review of hospital medical records, were invited to participate in the study. Patients were excluded if they were in an unstable clinical condition at enrollment, such as unstable angina, hemodynamic instability, severe arrhythmia or receiving oxygen therapy. These criteria ensure patient safety. Additionally, patients unable to comprehend spoken Urdu were excluded to facilitate effective communication for data collection.

### 2.3. Sample Size

The sample size was calculated using OpenEpi Version 3 to be 354, utilizing the formula: n = [Z^2^ × *p* × (1 − *p*) × design effect]/MoE^2^. where n represents the sample size, Z denotes the confidence level (1.96 for a 95% confidence level), and *p* signifies the prevalence of 0.29 [20]. The design effect was set at 1 due to the absence of cluster sampling, and the margin of error (MoE) was 0.05.

### 2.4. Sampling and Data Collection

Convenience sampling was used to recruit patients for this study from the coronary care units. Data were collected from 1 May to 20 September 2025, through in-person interviews conducted with patients in the coronary care unit, utilizing the Urdu language. The in-person interview procedure ensured item non-response. Each interview lasted from 20 to 30 min. The instruments, based on the conceptual framework guiding this study, included self-care measures, person-related measures, problem-related measures and environment-related measures described below [17].

### 2.5. Measurements

Self-care was assessed using the Self-Care of Coronary Heart Disease Inventory (SC-CHDI) [21]. This instrument contains three scales and 23 items, namely the Self-Care Maintenance Scale (9 items) that measures the frequency of actions focused on maintaining CHD stability (for example, keeping medical appointments). The Self-Care Monitoring Scale consisted of (8-items) which evaluates how often the patient monitors the signs and symptoms of CHD (for example, blood pressure monitoring); finally, the Self-care Management Scale comprises (6-items) that aim to evaluate how likely the patient responds to CHD symptoms with specific behaviors (for example, taking an Aspirin in case of CHD symptoms). Each item was rated on a 5-point scale from Never to Always. The score of each scale ranges between 0 and 100, with a higher score meaning better self-care. The SC-CHDI scales had Cronbach’s alpha reliability scores ranging between 0.76 and 0.87 in the American patients with CHD [21], and 0.96 in the Urdu version [22]. In the current study, it was 0.86. The Urdu version of the SC-CHDI was used for data collection.

Person-related measures include age, sex was collected as biological variable, marital status, education, body mass index, lifestyle, and cigarette smoking. All of these variables are known to influence CHD. Cardiac self-care self-efficacy was measured using the Self-Care Self-Efficacy Scale (SC-SES) [23]. The SC-SES was used in its Urdu form following a translation and back-translation process [24]. The Self-Efficacy Scale comprises 10 items that evaluate the extent to which patients feel confident in adopting self-care behavior (for example, persist in following the treatment plan even when difficult). Each item on the scale is rated from 1 = not confident to 5 = extremely confident. The total score ranges from 0 to 100, with a higher score meaning greater self-efficacy. A multinational study encompassing the United States, Hong Kong, Italy, and Brazil yielded Cronbach’s alpha coefficients of 0.93, 0.89, 0.92, and 0.90 for each respective country [23]. Cronbach’s alpha in the current study was 0.81.

Anxiety was measured using the Generalized Anxiety Disorder (GAD-7) screener-7 [25]. The GAD instrument comprises seven items (for example, worrying too much about different things), rated on a Likert scale from 0 to 3, with a total score ranging from 0 to 21; higher scores indicate higher anxiety levels. The instrument had an adequate reliability score of 0.92 for the Urdu version [26]. Depression was measured using the Patient Health Questionnaire-9 (PHQ-9) [27]. The PHQ-9 contains 9 items (for example, little interest or pleasure in doing things), rated on a four-point Likert scale, with scores ranging from 0 to 27. Higher scores indicate higher levels of depressive symptoms. A previous study reported a Cronbach’s Alpha of 0.91 in the Urdu version [28]. Comorbidity was measured using the Charlson Comorbidity Index (CCI) [29], which allocates scores for 19 diseases according to severity (for example, diabetes). The total score ranges from 0 to 37, with higher scores indicating severe comorbidities. CCI enhances prognostic estimation and improves the prediction of clinical outcomes [29].

Environment-related measures included rural or urban residency; religious engagement (low or high involvement in religious activities); carer preference (sex preference for assistance in self-care-related activities); accessibility to ambulance services; and mode of commuting, which is via personal or public transportation.

### 2.6. Analyses

All information obtained from the instruments was entered into the Statistical Package for Social Sciences (SPSS) v27.0. Double data entry was then performed to ensure data accuracy and to detect typographical errors. No missing data were observed due to the in-person interview procedure; therefore, all analyses were conducted using the full sample (n = 354). Data were screened for outliers and normal distribution. Data within a skewness range of −2 to +2 were considered to be normally distributed. Sociodemographic and clinical variables were reported as frequencies and percentages, and continuous variables were reported as means and standard deviations. The SC-CHDI and SC-SES items were analyzed using the mean and standard deviation. Item-level comparisons were conducted as exploratory analyses to examine potential sex differences across individual scale items. The total score of SC-CHDI scales and SC-SES was standardized on a scale of 0 to 100 [30]. A score ≥ 70 was considered adequate [31]. Sex differences were evaluated using the chi-square and independent *t*-test. Variables significantly associated with self-care (maintenance, monitoring, and management) were analyzed using multiple linear regression. Assumptions of multiple linear regression were checked. Linearity and homoscedasticity were evaluated using residual plots, normality with P–P plots, and independence of errors with the Durbin–Watson statistic. Multicollinearity was assessed using variance inflation factors (VIF). The variable residence was removed due to high multicollinearity (VIF = 6.2). The exclusion of residence did not alter the magnitude or direction of regression coefficients. Person-related variables associated with self-care were age, sex, marital status, education, cigarette smoking, body mass index, and lifestyle; problem-related variables were comorbidity index, anxiety, and depression; and finally, environment-related variables associated with self-care were identified by religious engagement, commuting mode, access to ambulance, and carer preference. A 95% level of significance was used for analyses.

## 3. Results

### 3.1. Characteristics of Sample Regarding Person, Problem and Environment Variables

Of the 640 individuals approached for data collection, 354 consented to participate in the study, resulting in a response rate of 55.3%. Of the 354 patients, 57.6% were men and 42.4% were women. Most of the men 56.4% and women 58.0% were married. More men 65.2% than women (57.3%) achieved a graduate education, while others achieved metric, primary, and a few had not received any formal education. The mean age of women (Mean 59.09, SD = 8.58 years) was significantly higher than that of men (Mean 56.75, SD = 10.11 years, *p* 0.03). The smoking prevalence was significantly higher among males 42.6% than among females 29.3% *p* 0.02. A significantly higher proportion of women 50% reported a sedentary lifestyle compared to men 31.38%, *p* < 0.03. The body mass index of women was significantly higher than that of men (Mean 27.50, SD = 5.31, Mean 26.29, SD = 4.68, respectively, *p* 0.02) (Table 1).

The depression score of men (Mean 16.29, SD = 1.20) was higher than that of women (Mean 15.95, SD = 1.65, *p* < 0.01). The Charlson comorbidity index of men was slightly higher than that of women (Mean 3.66, SD = 1.30; Mean 3.49, SD = 1.14; *p* 0.21). The anxiety score of women was significantly higher than that of men (Mean 15.17, SD = 2.98; Mean 13.11, SD = 3.51, *p* < 0.01). Most of the patients were urban residents (68.1% men and 64.7% women). Many patients commuted to the hospital through public transport (men, 31.9%; women, 35.3%). Men reported higher accessibility to ambulances (67.6%) than did women (61.3%). More than 71.3% of women engaged in religious activities compared to 66.2% of men (Table 1).

### 3.2. Adequacy Proportions for Self-Care Maintenance, Monitoring, Management, and Self-Efficacy

Just over half of both men (54.4%) and women (52.7%) demonstrated adequate self-care maintenance. However, self-care monitoring was poor, with only 2.0% of men and 8.7% of women achieving adequate levels. Women outperformed men in self-care management (36.0% vs. 17.2%). Self-care self-efficacy was comparable between sexes, with approximately half of the participants reporting adequate confidence Table 2.

### 3.3. Self-Care Maintenance, Monitoring and Management

Self-care maintenance scale mean score was marginally adequate (men 70.35, SD = 6.85; women 71.34, SD = 10.50). Overall, “try to avoid getting sick” had the highest mean score (Mean 4.27, SD = 0.86) and “doing physical activity” had the lowest mean score (Mean 2.87, SD = 0.70). Men performed higher self-care maintenance regarding doing physical activity and avoiding getting sick (*p* < 0.01). Whereas women performed significantly better self-care maintenance behaviors than men, such as “relieve stress”, and “avoid cigarettes and/or smokers” (*p* < 0.01), and “take prescribed medicines without missing a dose” (*p* 0.02).

The self-care monitoring scale mean scores were inadequate for both sexes (men 56.97, SD = 8.89; women 55.38, SD = 11.75). Overall, the highest mean score was recorded for “monitoring your condition” (Mean 3.86, SD = 0.54) and lowest for “symptom recognition” (Mean 1.68, SD = 1.05), and these were statistically significantly higher in women than in men (*p* < 0.01). In contrast, men performed significantly better self-care monitoring for blood pressure, weight, and symptoms (*p* < 0.01), and monitoring about getting tired during usual activities (*p* 0.04).

Self-care management scale mean scores were also inadequate (men 64.49, SD = 8.44; women 65.39, SD = 11.30). Overall, the highest mean score (Mean 4.72, SD = 0.52) was regarding “tell your healthcare provider about symptoms at next office visit” in which men performed significantly better than women (*p* < 0.01). The lowest mean score (Mean 1.74, SD = 0.77) was for “did treatment you used make you feel better,” in which women performed significantly better (*p* < 0.01). Moreover, women performed better when taking medications (*p* < 0.01). Men performed better in “calling healthcare providers for guidance” (*p* 0.02) (Table 3).

### 3.4. Self-Care Self-Efficacy

Women report higher self-care self-efficacy on multiple items including ‘keeping stable and symptom free’ (*p* < 0.01), following treatment plan (*p* 0.02), persistent in routine monitoring (*p* 0.04), recognizing changes in health (*p* < 0.01), persistence in finding remedy for symptoms (*p* 0.02), and evaluating how well a remedy worked (*p* < 0.01) (Table 4).

### 3.5. Person, Problem, and Environment-Related Variables Associated with Self-Care

A lack of formal education, having a sedentary lifestyle, depressive symptoms, less religious engagement, and commuting via public transport were significantly associated with lower self-care maintenance scores (*p* < 0.01). However, ambulance accessibility was associated with a higher self-care maintenance score (*p* < 0.03). Poor Self-Care Monitoring was significantly associated with lack of formal education, anxiety score, depression score and commuting via public transportation (*p* < 0.01), higher comorbidity score (*p* 0.02). Older age (*p* 0.02), higher depression score and commuting via public transport were significantly associated with lower Self-Care Management score (*p* < 0.01). However, being single was associated with higher Self-Care Management (*p* < 0.01). Depression and commuting via public transport were significantly associated across all self-care domains negatively (*p* < 0.01) (Table 5).

## 4. Discussion

This study examined factors associated with self-care in patients with CHD. The salient findings of this study indicate that self-care maintenance was marginally adequate, while self-care monitoring and management were inadequate. Depression and commuting via public transport emerged as a significant factor negatively associated with all self-care domains, including self-care maintenance, self-care monitoring and self-care management, with higher levels of depressive symptoms observed in men, whereas anxiety was higher among women.

Self-care maintenance was adequate in the present study. This finding is consistent with Thai [13], Indonesian [14], and Jordanian [32] studies that reported adequate self-care maintenance behaviors, including keeping appointments with healthcare providers and using stress-relieving strategies. However, a Pakistani study [22] and two European studies [31,33] reported lower self-care maintenance than that reported in the current study. In an Italian study, patients reported struggling with stress management, exhibiting low physical activity, and being less likely to choose low-fat options when dining out or visiting friends [31]. The current study found that most patients were engaged in religious activities that may reduce stress, and a higher proportion of women engaged in religious activities and stress-reduction strategies than men. Some studies have reported the positive influence of religious engagement on stress reduction [34]. The most inadequate self-care maintenance among women was asking for low-fat items when dining out or with friends, and engaging in physical activities. This reluctance for healthier food options may stem from peer pressure and can lead to increased consumption of high-fat meals, resulting in elevated cholesterol levels. At the same time, most men with inadequate self-care maintenance did not avoid cigarette smoking or being smokers. This behavior may stem from social pressures, as a stress relief, or a lack of awareness about the long-term consequences of smoking on their health. Another important finding of this study was that several variables were associated with inadequate self-care maintenance, including lack of formal education, less engagement in religious activities, the use of public transit for commuting, sedentary lifestyle, and depressive symptoms. Individuals with low confidence in their ability to manage their health are more likely to struggle with effective self-care practices. Age and comorbidities have also been recognized as associated factors in self-care [31]. Older individuals and those with comorbidities could face unique challenges in implementing self-care strategies, potentially due to physical limitations, cognitive decline, or managing multiple health issues simultaneously. Koson et al. identified that marital status and education were significantly associated with self-care [13]. Married individuals may benefit from spousal support in health management. However, the current study found an inverse relationship between marital status and self-care management. This unexpected finding may be context-specific, particularly for women who become overly dependent on their partners for self-care decisions, potentially diminishing their personal accountability. In contrast, individuals who are single may exercise greater personal autonomy in managing their self-care out of necessity. These findings underscore the multifaceted nature of self-care maintenance in CHD as well as significant disparities in variables across international studies, emphasizing the need for culturally sensitive and context-specific interventions rather than a one-size-fits-all approach to promoting self-care behaviors.

Of major concern is the finding that Self-Care Monitoring was inadequate and had the lowest scores among the SC-CHDI scales. This finding is inconsistent with those of Thai [13] and Jordan [32], in which patients reported adequate self-care monitoring on self-care subdomains. However, a Pakistani [22] and an Italian study shared similar findings [31], including checking blood pressure, monitoring side effects of medications, and monitoring weight. Men performed significantly better self-care monitoring regarding multiple aspects of self-care, including monitoring for blood pressure, weight, and monitoring whether getting tired during usual activities in the present study. In the Pakistani context, men may be able to monitor their health more consistently because they often have greater independence in seeking care and making health-related decisions. Greater mobility and easier access to healthcare facilities may also contribute to this pattern. Interestingly, women in this study recognized cardiac symptoms more quickly than men. The finding may suggest that women are more attentive to bodily changes. However, symptom recognition does not always translate into prompt healthcare seeking, particularly within sociocultural contexts where women may delay care due to limited autonomy. Therefore, while women in the present study appeared to identify symptoms earlier, further exploration is needed. Men had a significantly higher comorbidity index than women in the current study, which could present a greater challenge in recognizing the symptoms of comorbid conditions. A Thai study reported a negative impact of comorbidities on self-care behaviors in patients with CHD [13]. Lack of self-monitoring can affect an individual’s ability to recognize and respond to significant health-related changes, potentially affecting their overall well-being and health outcomes. This study identified that low levels of education, commuting through public transport, a higher comorbidity index, higher anxiety score, and higher depression score were associated with inadequate self-care monitoring. Studies report that adequate self-care monitoring was associated with social support factors and the younger age group [32], being married and higher education level [13], and a younger age and fewer comorbidities [31]. Self-care and patient-centered interventions can improve self-care monitoring [22].

Self-care management was also inadequate in this study. This finding is consistent with a Pakistani study [22] and international studies [13,31,33]. Overlapping inadequate self-care management behaviors in international and current studies include taking aspirin, taking medication for symptom management, and feelings about how well treatment works. A clinical trial found that the use of reminder applications can improve medication adherence behaviors in patients with CHD [35]. In the current study, men preferred to call health care providers for guidance. In contrast, women reported significantly better medication-related self-care management than men. This finding may reflect greater adherence to prescribed treatment regimens and a stronger sense of responsibility toward medication routines among female patients. A recent study reported that women adopt a more independent role in managing their health [36]. The lowest self-care behavior in the current study was that the treatment used made patients feel better. Significant variables associated with inadequate self-care management in this study were older age, commuting through public transport, and higher depression scores. Being single is positively linked with self-care management. Older age and comorbidity [13,31] are significantly associated with poor self-care management across international studies. However, married patients with social support [13,32] and a high level of education [13] were significantly associated with adequate self-care management [13,14]. The discrepancy between matrimonial status and self-care outcomes in this study, compared to previous research, needs to be further explored.

The study findings revealed that overall self-care self-efficacy scores were slightly above the adequacy threshold for men, but marginally below the adequate level for women. These findings are consistent with those of Italian and Jordanian studies [31,32] and among a diverse population with CHD [37]. However, inadequate self-care monitoring and self-care management do not align with an adequate SC-SES score. This discrepancy suggests that patients may overestimate their capacity to manage their condition effectively. They may possess sufficient knowledge about CHD but lack the requisite skills and resources for self-care monitoring and management.

Depressive symptoms and commuting via public transport have shown a significant inverse association across all self-care domains. A recent study reports that patients with depressive symptoms were less likely to engage in self-care activities [38]. Depressive symptoms could make it harder for patients to stay consistent with self-care behaviors. In Pakistan, where mental health concerns are often overlooked [39], depressive symptoms in cardiac patients may remain unaddressed, further affecting self-care. Patient-centered self-care interventions and psychological screening in routine cardiac care can alleviate depressive symptoms and improve self-care [22]. Commuting via public transport is considered a proxy variable for low resources. Long travel times, fatigue, financial strain, and overcrowded systems may limit patients’ ability to attend follow-ups. Research has indicated that insufficient transportation is associated with postponed or missed appointments, delayed access to healthcare services, and non-compliance with medication prescriptions [40]. Improving the affordability of public transportation promotes usage and health [41].

The study findings revealed that men presented with CHD at the age of 56.7 years, which is almost 2.4 years younger than that of women, with an average age of 59.1 years. The risk of CHD in women increases following menopause, and women typically develop CHD several years later than men do. The manifestation of CHD is delayed in women because of the protective effects of hormones prior to menopause [42]. This age is also much lower than the CHD studies in Iceland [33] and Italy [31] whose patients were older than 60 years. A higher proportion of men in the current study were smokers. Almost half of the women in the present study reported a sedentary lifestyle. The sedentary lifestyle among women is ubiquitous, but more prevalent in Eastern Mediterranean countries, including Pakistan [43]. A considerable proportion of the patients in this study were unmarried, divorced, or widowed. Studies across international populations have shown that unmarried individuals, including those divorced, separated, widowed, or single, experience a higher incidence of unfavorable cardiovascular events than their married peers [44]. However, the quality of marital relationships affects cardiovascular health. Matrimony may offer greater protective benefits to men than to women [44].

Limitations include its cross-sectional design, which restricts causal inferences about variable relationships, and the use of convenience sampling. Self-reported data during hospitalization may be subject to recall and social desirability bias. A limitation of this study is potential nonresponse bias, as nonparticipants may differ from respondents, affecting generalizability. As a single-center study conducted in a private hospital, it limits generalizability and the exploration of cultural factors that may influence self-care behaviors. Despite these limitations, this study had several strengths. The study had an adequate sample size with balanced representation of men and women. The study employed validated instruments and a theory-based approach to self-care, integrating person, clinical, and environmental variables within an ecological model, offering a comprehensive assessment of self-care that few other studies have considered.

Healthcare policies should focus on improving self-care education and support for patients with CHD, particularly in the monitoring and management areas where scores are inadequate. Screening for depressive symptoms should be integrated into routine cardiac care for early identification and mental health referral. Patient-centered self-care interventions should focus on sedentary behaviors in women and on lowering smoking rates in men. Policies should also address social factors affecting self-care, including access to healthcare and commuting services.

## 5. Conclusions

In conclusion, the findings of this study reveal that self-care monitoring, self-care management, and self-efficacy were suboptimal among patients. This study identified several variables associated with self-care behaviors. Depressive symptoms and commuting via public transport were negatively associated across all self-care domains, including maintenance, monitoring and management. Sex-specific factors associated with worse self-care included depression, smoking, and multiple comorbidities in men, and anxiety and sedentary behavior in women. Whereas being single and having access to an ambulance improved self-care. These findings highlight the complex interplay between person, environment, problem-related variables, and individual confidence in engaging in effective self-care. Future research should evaluate patient-centered self-care interventions to enhance self-care practices and employ cohort designs to identify sociocultural and contextual factors influencing self-care behaviors.

## Figures and Tables

**Table 1:** Person, problem and environment-related description of patients by sex (n = 354).

Person-Related Variables		Men (n = 204)		Women (n = 150)		Total (n = 354)		*p*-Value
		%	n	%	n	%	n	
Marital status	Married	56.4	115	58.0	87	57.1	202	0.07
	Unmarried	27.9	57	27.3	41	27.7	98	
	Divorced	2.9	6	1.3	2	2.3	8	
	Widow/widower	12.7	26	13.3	20	13.0	46	
Education	Uneducated	4.4	9	3.3	5	4.0	14	0.12
	Primary	6.9	14	9.3	14	7.9	28	
	Metric	23.5	48	30.0	45	26.3	93	
	Graduate	65.2	133	57.3	86	61.9	219	
Cigarette Smoking **	Yes	42.6	87	29.3	44	37.0	131	0.02 *
Lifestyle **	Active	68.62	140	50.0	75	54.0	191	0.03 *
	Sedentary	31.38	64	50.0	75	46.0	163	
Age * (Mean, SD)		56.75, 10.11		59.09, 8.58		57.74, 9.55		0.03 *
Body Mass Index * (Mean, SD)		26.29, 4.68		27.50, 5.31		26.96, 5.20		0.02 *
**Problem-related variables**								
Comorbidity Index (Mean, SD)		3.66, 1.30		3.49, 1.14		3.59, 1.24		0.21
Anxiety score * (Mean, SD)		13.11, 3.51		15.17, 2.98		13.98, 3.45		<0.01 *
Depression score * (Mean, SD)		16.29, 1.20		15.95, 1.65		16.15, 1.41		<0.01 *
**Environment-related variables**		%	n	%	n	%	n	
Residence	Urban	68.1	139	64.7	97	66.7	236	0.23
	Rural	31.9	65	35.3	53	33.3	118	
Commuting mode	Own	68.1	139	64.7	97	66.7	236	0.13
	Public	31.9	65	35.3	53	33.3	118	
Ambulance Access	Yes	67.6	138	61.3	92	65.0	230	0.51
Carer Preference	Yes	46.6	95	42.0	63	44.6	158	0.34
Religious engagement	High engaged	66.2	135	71.3	107	68.4	242	0.67
	Low engaged	33.8	69	28.7	43	31.6	112	

SD standard deviation, * independent *t*-test significant *p*-value < 0.05, ** Chi-square.

**Table 2:** Proportions of self-care and self-efficacy adequacy by sex in patients (n = 354).

Self-Care Scales	Men (n = 204)						Women (n = 150)					
	Inadequate			Adequate			Inadequate			Adequate		
	%	n	95% CI (Lower, Upper)	%	n	95% CI (Lower, Upper)	%	n	95% CI (Lower, Upper)	%	n	95% CI (Lower, Upper)
Maintenance	45.6	93	0.38, 0.53	54.4	111	0.47, 0.61	47.3	71	0.39, 0.55	52.7	79	0.44, 0.60
Monitoring	98.0	200	0.95, 0.99	2.0	4	0.00, 0.04	91.3	137	0.85, 0.95	8.7	13	0.04, 0.14
Management	82.8	169	0.77, 0.88	17.2	35	0.12, 0.23	64.0	96	0.56, 0.71	36.0	54	0.28, 0.44
Self-Efficacy	50.5	103	0.43, 0.57	49.5	101	0.42, 0.56	54.0	81	0.45, 0.62	46.0	69	0.37, 0.54

CI confidence interval.

**Table 3:** Self-Care of Coronary Heart Disease Inventory scores by sex and overall (n = 354).

Self-Care Maintenance (Total Score and Items’ Scores)	Men (n = 204)		Women (n = 150)		Total (n = 354)		*p*-Value
	Mean	SD	Mean	SD	Mean	SD	
Self-Care Maintenance (total score)	70.35	6.85	71.34	10.50	70.77	8.59	0.95
Self-Care Maintenance (items’ score)							
1. Keep appointments with the healthcare provider	3.89	0.94	3.95	1.05	3.91	0.98	0.57
2. Take aspirin or other blood thinner	3.52	0.80	3.35	1.00	3.45	0.90	0.08
3. Do something to relieve stress (e.g., medication, yoga, music)	3.66	0.53	3.89	0.73	3.76	0.63	<0.01
4. Do physical activity (e.g., take a brisk walk, use the stairs)	3.09	0.63	2.58	0.69	2.87	0.70	<0.01
5. Take prescribed medicines without missing a dose	3.29	0.81	3.49	0.78	3.38	0.80	0.02
6. Ask for low-fat items when eating out or visiting others	3.00	0.82	2.79	0.85	2.92	0.84	0.09
7. Try to avoid getting sick (e.g., flu shot, washing hands)	4.40	0.81	4.09	0.91	4.27	0.86	<0.01
8. Eat fruits and vegetables	4.21	0.68	4.28	0.80	4.24	0.73	0.37
9. Avoid cigarettes and/or smokers	2.60	0.95	3.26	1.27	2.88	1.14	<0.01
Self-Care Monitoring (total score)	56.97	8.89	55.38	11.75	56.29	10.22	0.67
Self-Care Monitoring (items’ score)							
10. Monitor your condition	3.76	0.57	4.00	0.48	3.86	0.54	<0.01
11. Pay attention to changes in how you feel	3.75	0.52	3.90	0.38	3.81	0.47	<0.01
12. Check your blood pressure	3.18	0.85	2.81	1.28	3.02	1.07	<0.01
13. Monitor whether you get tired more than usual doing normal activities	3.28	0.67	3.11	0.95	3.21	0.81	0.04
14. Monitor for medication side-effects	2.01	0.57	2.06	0.71	2.03	0.63	0.46
15. Monitor for symptoms	2.33	0.74	1.99	0.60	2.18	0.70	<0.01
16. Monitor your weight	2.93	1.00	2.44	0.96	2.72	1.01	<0.01
17. Symptom recognition	1.56	0.86	1.85	1.25	1.68	1.05	<0.01
Self-Care Management (total score)	64.49	8.44	65.39	11.30	64.87	9.75	0.99
Self-Care Management (items’ score)							
18. Change your activity level (slow down, rest)	3.70	0.68	3.80	1.08	3.74	0.87	0.27
19. Take an aspirin	3.41	0.65	3.64	0.63	3.51	0.65	<0.01
20. Take a medicine to make the symptoms decrease or go away	3.37	0.68	3.51	0.58	3.43	0.64	0.04
21. Call a healthcare provider for guidance	2.47	1.28	2.14	1.38	2.33	1.33	0.02
22. Tell the healthcare provider about the symptoms at the next visit	4.78	0.47	4.63	0.57	4.72	0.52	<0.01
23. Did the treatment you used make you feel better	1.63	0.66	1.90	0.89	1.74	0.77	<0.01

Note: SD standard deviation, all comparisons between men and women were performed with the *t*-test for independent samples.

**Table 4:** Self-care self-efficacy in patients with CHD by sex (n = 354).

Self-Care Self-Efficacy (Total Score and Items’ Score)	Men (n = 204)		Women (n = 150)		Total (n = 354)		*p*-Value
	Mean	SD	Mean	SD	Mean	SD	
Self-Care Self-Efficacy (total score)	71.47	6.27	68.86	7.81	69.97	7.00	0.79
Self-Care Self-Efficacy (items’ score)							
1. Keep yourself stable and free of symptoms?	3.77	0.95	4.12	0.89	3.92	0.94	<0.01
2. Follow the treatment plan you have been given?	3.90	0.52	4.03	0.65	3.95	0.58	0.02
3. Persist in following the treatment plan even when difficult?	3.01	0.70	2.98	0.81	3.00	0.75	0.71
4. Monitor your condition routinely?	3.61	0.55	3.64	0.55	3.62	0.55	0.64
5. Persist in routine monitoring condition even when difficult?	2.80	0.63	2.95	0.74	2.86	0.68	0.04
6. Recognize changes in your health if they occur?	3.20	0.47	3.42	0.67	3.29	0.57	<0.01
7. Evaluate the importance of your symptoms?	3.78	0.59	3.83	0.58	3.80	0.58	0.50
8. Do something to relieve your symptoms?	4.26	0.73	4.44	0.67	4.34	0.71	0.12
9. Persist in finding a remedy for symptoms even when difficult?	2.97	0.72	2.99	0.94	2.98	0.82	0.02
10. Evaluate how well a remedy works?	3.12	0.41	3.34	0.57	3.21	0.49	<0.01

Note: SD standard deviation, all comparisons between men and women were performed with the *t*-test for independent samples.

**Table 5:** Variables associated with self-care maintenance, monitoring and management among patients with CHD (n = 354).

Variables			Self-Care Maintenance					Self-Care Monitoring					Self-Care Management			
		t	OR	CI 95%	*p*-Value		t	OR	CI 95%	*p*-Value		t	OR	CI 95%	*p*-Value	
Person-related	Age	0.04	0.01	0.08, 0.15	0.97	**Durbin Watson Statistics 1.88, R^2^ = 0.33**	0.39	0.02	0.08, 0.12	0.67	**Durbin Watson Statistics 1.78, R^2^ = 0.30**	−2.66	−0.15	−0.25, −0.05	0.02	**Durbin Watson Statistics 1.55, R^2^ = 0.18**
	Sex	0.58	0.64	0.03, 1.26	0.56		0.86	0.05	1.22, 3.12	0.39		0.21	0.31	0.01, 2.46	0.84	
	Marital status	1.43	0.07	−0.46, 2.91	0.15		1.28	0.06	0.71, 3.38	0.20		5.38	5.77	3.66, 7.88	<0.01	
	Education	−2.75	−0.16	−4.62, −0.77	<0.01		−2.48	−0.14	−5.29, −0.61	<0.01		−1.03	−0.06	−3.67, 1.15	0.31	
	Cigarette smoking	−1.57	−0.08	−3.20, 0.18	0.12		0.12	0.01	−2.04, 2.29	0.90		0.55	0.03	−1.61, 2.85	0.58	
	Body mass index	0.43	0.02	−0.12, 0.18	0.67		0.81	0.04	0.01, 0.26	0.42		0.40	0.02	−0.15, 0.22	0.69	
	Lifestyle	−2.90	−0.14	−3.92, −0.75	<0.01		−0.20	−0.01	−2.12, 1.73	0.84		0.02	0.00	−1.96, 2.01	0.98	
Problem-related	Comorbidities	−1.28	−0.06	−1.10, 0.23	0.20		2.11	0.18	0.51, 1.11	0.02		−0.75	−0.04	−1.16, 0.52	0.45	
	Anxiety	−1.54	−0.07	−0.43, 0.05	0.12		−5.16	−0.26	−1.05, −0.47	<0.01		−1.42	−0.08	−0.51, 0.08	0.16	
	Depression	−6.28	−0.31	−2.67, −1.40	<0.01		−6.19	−0.31	−3.21, −1.66	0.01		−4.86	−0.27	−2.77, −1.17	<0.01	
Environment-related	Religious engagement	−2.66	−0.13	−4.22, −0.63	<0.01		−1.91	−0.10	−4.29, 0.06	0.06		−1.82	−0.10	−4.32, 0.17	0.07	
	Commuting mode	−5.20	−0.45	−11.18, −5.79	<0.01		−4.50	−0.39	−12.25, −4.80	<0.01		−2.54	−0.24	−8.81, −1.12	0.01	
	Ambulance access	2.19	3.46	1.13, 5.79	0.03		1.69	0.13	0.47, 6.20	0.09		0.47	0.04	2.62, 4.26	0.64	
	Carer preference	−0.25	−0.01	−2.25, 1.73	0.80		0.05	0.00	−2.36, 2.47	0.96		0.88	0.06	1.38, 3.60	0.38	

Note: Analysis performed with multiple linear regression,; CI confidence interval, OR odds ratio, CHD coronary heart disease, [reference groups; (Sex; Men), (marital status; single), (educational level; low education level), (Cigarette smoking; yes), (lifestyle; sedentary), (religious engagement; low), (commuting mode; public transportation), (ambulance accessibility; no), (carer preference; yes]; age, BMI, anxiety, depression and comorbidity index were continuous variables.

## Data Availability

The data are available upon reasonable request because the data are not publicly available due to privacy and ethical restrictions.

## Abbreviations

The following abbreviations are used in this manuscript:

CHD	Coronary heart disease
WHO	World Health Organization
IRB	Institutional Review Board
SPSS	Statistical Package for Social Sciences
SC	Self-care
CHDI	Coronary Heart Disease Inventory
SES	Self-Efficacy Scale
GAD	Generalized Anxiety Disorder
PHQ	Patient Health Questionnaire
CCI	Charlson Comorbidity Index
SD	Standard Deviation

## References

[1] National Center for Health Statistics (2023). Multiple Cause of Death 2018–2021 on CDC WONDER Database.

[2] Tsao C.W., Aday A.W., Almarzooq Z.I., Anderson C.A.M., Arora P., Avery C.L., Avery C.L., Baker-Smith C.M., Beaton A.Z., Boehme A.K. (2023). Heart Disease and Stroke Statistics—2023 Update: A Report from the American Heart Association. Circulation.

[3] World Health Organization (2026). Cardiovascular Diseases (CVDs). https://www.who.int/news-room/fact-sheets/detail/cardiovascular-diseases-(cvds).

[4] Gaziano T.A., Bitton A., Anand S., Abrahams-Gessel S., Murphy A. (2010). Growing epidemic of coronary heart disease in low- and middle-income countries. Curr. Probl. Cardiol..

[5] Razzak H.A., Harbi A., Shelpai W., Qawas A. (2017). Prevalence and risk factors of cardiovascular disease in the United Arab Emirates. Hamdan Med. J..

[6] Suliman A. (2011). The state of heart disease in Sudan. Cardiovasc. J. Afr..

[7] Roth G.A., Mensah G.A., Johnson C.O., Addolorato G., Ammirati E., Baddour L.M., Barengo N.C., Beaton A.Z., Benjamin E.J., Benziger C.P. (2020). Global Burden of Cardiovascular Diseases and Risk Factors, 1990–2019: Update From the GBD 2019 Study. J. Am. Coll. Cardiol..

[8] Garcia M., Mulvagh S.L., Merz C.N.B., Buring J.E., Manson J.E. (2016). Cardiovascular Disease in Women: Clinical Perspectives. Circ. Res..

[9] World Heart Federation (2023). World Heart Report 2023: Confronting the World’s Number One Killer.

[10] Dattani S., Samborska V., Ritchie H., Roser M. (2023). Cardiovascular Diseases. https://ourworldindata.org/cardiovascular-diseases.

[11] Samad Z., Hanif B. (2023). Cardiovascular Diseases in Pakistan: Imagining a Postpandemic, Postconflict Future. Circulation.

[12] Simonelli N., Bolgeo T., Iovino P., Di Matteo R., Maconi A., Vellone E. (2023). Self-care in coronary heart disease patient and caregiver dyads (HEARTS-IN-DYADS)—Protocol of a multicenter longitudinal study. Res. Nurs. Health.

[13] Koson N., Srisuk N., Juntratip N., Borvornsudhasin P., Thompson D.R., Ski C.F. (2025). Clinical and demographic moderators of self-care and hospitalizations in pre-coronary artery bypass grafting patients: A cross-sectional study. J. Clin. Nurs..

[14] Mailani F., Nurikhsani N., Muthia R. (2023). Illness Perception and Self-Care in Coronary Heart Disease Patients: A Cross-Sectional Study. Caring Indones. J. Nurs. Sci..

[15] Victor G., Shishani K., Vellone E., Froelicher E.S. (2025). The Global Burden of Cardiovascular Disease in Adults: A Mapping Review. J. Cardiovasc. Nurs..

[16] Regitz-Zagrosek V., Gebhard C. (2023). Gender medicine: Effects of sex and gender on cardiovascular disease manifestation and outcomes. Nat. Rev. Cardiol..

[17] Riegel B., Dickson V.V., Vellone E. (2022). The Situation-Specific Theory of Heart Failure Self-care: An Update on the Problem, Person, and Environmental Factors Influencing Heart Failure Self-care. J. Cardiovasc. Nurs..

[18] Sharma A., Minh Duc N.T., Luu Lam Thang T., Nam N.H., Ng S.J., Abbas K.S., Huy N.T., Marušić A., Paul C.L., Kwok J. (2021). A Consensus-Based Checklist for Reporting of Survey Studies (CROSS). J. Gen. Intern. Med..

[19] Thygesen K., Alpert J.S., Jaffe A.S., Chaitman B.R., Bax J.J., Morrow D.A., White H.D. (2018). Fourth Universal Definition of Myocardial Infarction (2018). Circulation.

[20] Osteresch R., Fach A., Schmucker J., Eitel I., Langer H., Hambrecht R., Wienbergen H. (2019). Long-Term Risk Factor Control After Myocardial Infarction-A Need for Better Prevention Programmes. J. Clin. Med..

[21] Vaughan Dickson V., Lee C.S., Yehle K.S., Mola A., Faulkner K.M., Riegel B. (2017). Psychometric Testing of the Self-Care of Coronary Heart Disease Inventory (SC-CHDI). Res. Nurs. Health.

[22] Perveen R., Saqib M., Kausar S., Asmat K. (2025). Feasibility and preliminary effectiveness of a patient-centered self-care intervention for adults with coronary heart disease in a low resource setting. Prev. Med. Rep..

[23] Yu D.S.F., De Maria M., Barbaranelli C., Vellone E., Matarese M., Ausili D., Rejane R.-S.E., Osokpo O.H., Riegel B. (2021). Cross-cultural applicability of the Self-Care Self-Efficacy Scale in a multi-national study. J. Adv. Nurs..

[24] Perveen R., Asmat K., Victor G. (2025). Assessing the Psychometric Properties of the Urdu Version of the Self-Care Coronary Heart Disease Inventory v3.0 and Self-Care Self-Efficacy Scale.

[25] Löwe B., Decker O., Müller S., Brähler E., Schellberg D., Herzog W., Herzberg P.Y. (2008). Validation and standardization of the Generalized Anxiety Disorder Screener (GAD-7) in the general population. Med. Care.

[26] Ahmad S., Hussain S., Shah F.S., Akhtar F. (2017). Urdu translation and validation of GAD-7: A screening and rating tool for anxiety symptoms in primary health care. J. Pak. Med. Assoc..

[27] Kroenke K., Spitzer R.L., Williams J.B. (2001). The PHQ-9: Validity of a brief depression severity measure. J. Gen. Intern. Med..

[28] Ahmad S., Hussain S., Akhtar F., Shah F.S. (2018). Urdu translation and validation of PHQ-9, a reliable identification, severity and treatment outcome tool for depression. J. Pak. Med. Assoc..

[29] Charlson M.E., Carrozzino D., Guidi J., Patierno C. (2022). Charlson Comorbidity Index: A Critical Review of Clinimetric Properties. Psychother. Psychosom..

[30] Riegel B., De Maria M., Barbaranelli C., Matarese M., Ausili D., Stromberg A., Vellone E., Jaarsma T. (2022). Symptom Recognition as a Mediator in the Self-Care of Chronic Illness. Front. Public Health.

[31] Di Matteo R., Bolgeo T., Dal Molin A., Bassola B., Lusignani M., Maconi A., Rasero L., Vellone E., Iovino P. (2025). Self-care behaviours and their determinants in people affected by coronary heart disease. J. Clin. Nurs..

[32] Abdelhameed O., Nemer M. (2022). Self-efficacy and Self-care Behaviors among Patients with Coronary Artery Disease in Jordan. Int. Res. J. Pharm. Med. Sci..

[33] Ingadóttir B., Svavarsdóttir M.H., Jurgens C.Y., Lee C.S. (2024). Self-care trajectories of patients with coronary heart disease: A longitudinal, observational study. Eur. J. Cardiovasc. Nurs..

[34] Abu H.O., Mcmanus D.D., Lessard D.M., Kiefe C.I., Goldberg R.J. (2019). Religious practices and changes in health- related quality of life after hospital discharge for an acute coronary syndrome. Health Qual. Life Outcomes.

[35] Santo K., Singleton A., Rogers K., Thiagalingam A., Chalmers J., Chow C.K., Redfern J. (2019). Medication reminder applications to improve adherence in coronary heart disease: A randomised clinical trial. Heart.

[36] Llubes-Arrià L., Briones-Vozmediano E., Mateos J.T., Sol-Cullere J., Gea-Sánchez M., Rubinat-Arnaldo E. (2025). Gender differences in self-management activation among patients with multiple chronic diseases: A qualitative study. Arch. Public Health.

[37] Sugiharto F., Nuraeni A., Trisyani Y., Putri A.M., Armansyah N.A., Zamroni A.H. (2023). A Scoping Review of Predictors Associated with Self-Efficacy Among Patients with Coronary Heart Disease. Vasc. Health Risk Manag..

[38] Buck H.G., Jurgens C.Y., Spintzyk E., Doyle J., Bellfield T., Babicheva V., Chen S., Buonafede G., Solaimani M., Lam L. (2026). Psychological self-care in chronic illness: A cross-sectional mixed-methods examination of strategies used by adults with cardiovascular disease. Eur. J. Cardiovasc. Nurs..

[39] Malik M., Rehman H., Hussain A., Hashmi A., Al-Sunaidar K.A., Balogh G., Gajdács M., Jamshed S. (2025). Psychological Burden and Coping Strategies Among Pakistani Adults: A Cross-Sectional Survey Study. Epidemiologia.

[40] Ibeh C.C., Fuller D., Alhassan J.A.K. (2025). The health and socioeconomic impacts of free transportation initiatives: A scoping review. J. Transp. Health.

[41] Alhassan J.A.K., Fuller D., Woytowich R. (2025). The promises and perils of a free rural inter-city transportation scheme: A mixed-methods study from Northern Saskatchewan. Can. J. Public Health.

[42] El Khoudary S.R., Aggarwal B., Beckie T.M., Hodis H.N., Johnson A.E., Langer R.D., Limacher M.C., Manson J.E., Stefanick M.L., Allison M.A. (2020). Menopause Transition and Cardiovascular Disease Risk: Implications for Timing of Early Prevention: A Scientific Statement From the American Heart Association. Circulation.

[43] Turk-Adawi K., Sarrafzadegan N., Fadhil I., Taubert K., Sadeghi M., Wenger N.K., Tan N.S., Grace S.L. (2018). Cardiovascular disease in the Eastern Mediterranean region: Epidemiology and risk factor burden. Nat. Rev. Cardiol..

[44] Dhindsa D.S., Khambhati J., Schultz W.M., Tahhan A.S., Quyyumi A.A. (2020). Marital status and outcomes in patients with cardiovascular disease. Trends Cardiovasc. Med..

[45] World Medical Association (2001). World Medical Association Declaration of Helsinki. Ethical principles for medical research involving human subjects. Bull. World Health Organ..

